# Effect of Parkin on methamphetamine‐induced α‐synuclein degradation dysfunction *in vitro* and *in vivo*

**DOI:** 10.1002/brb3.1574

**Published:** 2020-02-21

**Authors:** Yunle Meng, Honghua Qiao, Jiuyang Ding, Yitong He, Haoling Fan, Chen Li, Pingming Qiu

**Affiliations:** ^1^ School of Forensic Medicine Southern Medical University Guangzhou China; ^2^ Guangdong HuaTian Forensic Biology Judicial Evaluation Institute Qingyuan China; ^3^ School of Basic Medicine and Life Science Hainan Medical University Haikou China

**Keywords:** methamphetamine, neurotoxicity, Parkin, α‐syn

## Abstract

**Introduction:**

Methamphetamine (METH) is a psychostimulant drug with complicated neurotoxicity, and abuse of METH is very common. Studies have shown that METH exposure causes alpha‐synuclein (α‐syn) accumulation. However, the mechanism of α‐syn accumulation has not been determined.

**Methods:**

In this study, we established cell and animal models of METH intoxication to evaluate how METH affects α‐syn expression. In addition, to explore METH‐induced neurotoxicity, we measured the level of Parkin and the phosphorylation levels of α‐syn, Polo‐like kinase 2 (PLK2), the proteasome activity marker CD3δ, and the apoptosis‐related proteins Caspase‐3 and PARP. Parkin is a key enzyme in the ubiquitin–proteasome system. In addition, the effect of Parkin on METH‐induced neurotoxicity was investigated by overexpressing it in vitro and in vivo*.*

**Results:**

METH exposure increased polyubiquitin and α‐syn expression, as did MG132. Furthermore, the level of Parkin and the interaction between Parkin and α‐syn decreased after METH exposure. Importantly, the increases in α‐syn expression and neurotoxicity were relieved by Parkin overexpression.

**Conclusions:**

By establishing stable cell lines and animal models that overexpress Parkin, we confirmed Parkin as an important factor in METH‐induced α‐syn degradation dysfunction in vitro and in vivo. Parkin may be a promising target for the treatment of METH‐induced neurotoxicity.

## Graphical abstract

We verify that Parkin, as the E3 ubiquitin ligase, plays a critical role in METH‐induced degradation dysfunction of α‐syn in vitro and in vivo. The overexpression of Parkin significantly promotes the degradation of α‐syn and relieves METH‐induced neurotoxicity.

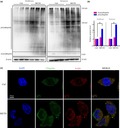

## 1 INTRODUCTION

Methamphetamine (METH) is a kind of amphetamine‐typed stimulant with complicated neurotoxicity that is widely abused around the world (Carvalho et al., 2012). METH is toxic to all systems of the body, especially the central nervous system (Yang et al., 2018). Accumulating evidence shows that METH induces apoptosis, oxidative stress, and mitochondrial dysfunction in vivo and in vitro (Du et al., 2018; Foroughi, Khaksari, Rahmati, Bitaraf, & Shayannia, 2019; Shin et al., 2018; Xu et al., 2018). Furthermore, METH can lead to the damage of dopaminergic neurons and Parkinson‐like pathology (Biagioni et al., 2019; Li et al., 2017). Despite much evidence of METH‐induced neuronal damage, the molecular mechanisms of METH neurotoxicity have not been completely revealed.

Alpha‐synuclein (α‐syn), composed of 140 amino acids, is expressed in the presynaptic and perinuclear regions of the central nervous system (Meade, Fairlie, & Mason, 2019). Studies have shown that under normal physiological conditions, α‐syn is low‐expression, soluble protein associated with dopamine uptake, synaptic plasticity, and vesicle maintenance (Sulzer & Edwards, 2019). However, under pathological conditions, α‐syn can undergo abnormal expression and form β‐sheet oligomers called protofibrils, which can develop into fibrils, fibers, oligomers, and other forms (Alam, Bousset, Melki, & Otzen, 2019; Henderson, Trojanowski, & Lee, 2019). Recent studies have shown that excessive expression of α‐syn has cytotoxic effects on neurons (Luna et al., 2018; Wegrzynowicz et al., 2019). Lewy bodies (LBs) have been shown to be composed mainly of α‐syn and are characteristic pathological signs of neurodegenerative diseases (Spillantini et al., 1997; Wu et al., 2019). Therefore, α‐syn is considered to be a protein closely related to neurodegenerative diseases (e.g., Parkinson's disease [PD] and Alzheimer's disease [AD]) (Henderson et al., 2019; Twohig & Nielsen, 2019). Previous studies, including studies by our research group, have found that α‐syn expression and the degree of cell injury increase with increasing METH concentrations (Biagioni et al., 2019; Qiao et al., 2019; Sun et al., 2019; Wang et al., 2017; Zhu et al., 2018).

Parkin is an extremely conserved protein and an E3 ubiquitin ligase that degrades proteins of the ubiquitin–proteasome system (UPS) (Martinez, Ramirez, Osinalde, Arizmendi, & Mayor, 2018). Parkin in cooperation with E1 activating enzyme and E2 binding enzyme promotes the ubiquitination of substrate proteins to be degraded (Corsa et al., 2019). Deletion or functional defects of Parkin may lead to the accumulation of substrate proteins that cannot be efficiently degraded (Brahmachari et al., 2019). Previous studies have found that α‐syn is a substrate protein of Parkin (Liu, Hebron, Shi, Lonskaya, & Moussa, 2019). In addition, the UPS plays a vital role in the degradation of α‐syn (Yuan et al., 2019). However, the contribution of Parkin to the degradation of α‐syn following METH‐induced α‐syn accumulation is unclear.

The study aimed to determine the effect of Parkin on α‐syn degradation dysfunction after METH exposure in vitro and in vivo*.* First, we tested whether impairment of the UPS pathway and Parkin level contributes to the dysfunction of α‐syn degradation after METH exposure. We measured α‐syn and polyubiquitin expression after exposure to METH and MG132 (a proteasome inhibitor). Then, we established cell and animal models of METH intoxication to investigate how METH affects Parkin and α‐syn expression and their interaction. In addition, the phosphorylation levels of α‐syn (P‐α‐syn), Polo‐like kinase 2 (PLK2), proteasome activity marker CD3δ, and the apoptosis‐related proteins Caspase‐3 and PARP were measured to detect METH‐induced neurotoxicity. Furthermore, a thorough investigation of the effect of Parkin on α‐syn degradation dysfunction was conducted by overexpressing Parkin in vitro and in vivo*.* The results indicated that METH can increase polyubiquitin and α‐syn expression, as can MG132. Furthermore, the level of Parkin and the interaction between Parkin and α‐syn were found to be decreased after METH exposure. Importantly, the increases in α‐syn and neurotoxicity were relieved after overexpressing Parkin. By establishing stable cell lines and animal models that overexpress Parkin, we confirmed Parkin as an important factor in α‐syn degradation dysfunction after METH exposure in vitro and in vivo.

## 2 MATERIALS AND METHODS

### 2.1 Animal protocol

Mice (C57 BL/6) were obtained from Laboratory Animal Center of Southern Medical University (Guangzhou, China) and housed in a dedicated animal room. The mice were divided randomly into two groups: a control group and a subacute METH exposure group (*n* = 3/group). Mice in the subacute exposure group received intraperitoneal (i.p.) injections at 12‐hr intervals for 4 days with 15 mg/kg METH diluted with saline (>99% purity; National Institutes for Food and Drug Control, Guangzhou, China). Mice in the control group received an equivalent amount of saline. We selected this exposure paradigm after referring to the concentrations of short‐term METH exposure in humans (Du et al., 2017). At 24 hr after the last injection, the mice were euthanized, and midbrain and striatum tissues of brain were dissected. The midbrain and striatum were selected based on our previous trials and a previous study (Jakowec, Donaldson, Barba, & Petzinger, 2001). The Institutional Animal Care and Use Committee at Southern Medical University approved the animal experiments, and the experiments were performed according to the guidelines of the National Institutes of Health (NIH).

### 2.2 Cell culture

SH‐SY5Y human neuroblastoma cells were used to investigate METH toxicity in vitro (Chen, Huang, Wang, Qiu, & Liu, 2013; Ma et al., 2019; Xicoy, Wieringa, & Martens, 2017). Cells were cultured in DMEM/F12 (1:1) with 10% fetal bovine serum (FBS; Gibco) and placed in a cell incubator in an environment of 5% carbon dioxide, a constant temperature of 37°C, and constant humidity.

Upon reaching approximately 80%–90% confluence, cells were exposed to a METH concentration of 1.0, 1.5, 2.0, 2.5, or 3.0 mM for 24 hr. Other cells were exposed to 2.0 mM METH for 0, 2, 6, 8, 12, or 24 hr. These concentrations were selected based on our experimental results and previous research (Ferrucci et al., 2017; Huang et al., 2015; Li et al., 2017).

### 2.3 Immunofluorescence analysis

We performed immunofluorescence labeling to determine ubiquitin and α‐syn expression levels in SH‐SY5Y cells. Cells were fixed in 4% paraformaldehyde and then washed three times with phosphate‐buffered saline (PBS). The cells were then blocked with 10% BSA containing 0.05% Triton X‐100 at room temperature for 30 min. Next, the cells were incubated with anti‐Ubiquitin (1:200, Abcam, Cat. #ab7780), anti‐α‐syn (1:200, CST, Cat. #4179), and anti‐Parkin antibody (1:200, Abcam, Cat. #ab77924) at 4°C overnight. Then, the cells were incubated with secondary antibody for 1 hr at room temperature. DAPI (H‐1200; Vector) was used to stain the nuclei. Images were obtained via a fluorescence microscope or a N‐SIM Nikon Super‐Resolution Microscope (Nikon).

### 2.4 Immunohistochemistry analysis

The brains were fixed in 4% paraformaldehyde and embedded in paraffin. Sections (3 μm in thickness) were made, pretreated in citrate buffer (0.01 M, pH 6.0) for antigen retrieval via hydrated autoclaving in a humid atmosphere for 10 min, and then blocked at room temperature with goat serum for 30 min. Then, the sections were incubated with the primary antibody anti‐Parkin antibody (1:200, Abcam, Cat. #ab77924) overnight at 4°C. 3,3′‐diaminobenzidine (DAB) kits (CW Bio, Cat. #CW2069) were used to develop the slices. Images were obtained using Zeiss Metasystem (Zeiss).

### 2.5 Western blot

Protease inhibitors were used to extract the protein samples in radioimmunoprecipitation assay (RIPA) buffer, and the BCA‐100 Protein Quantitative Analysis Kit (Biocolors) was used to detect protein concentration. Sodium dodecyl sulfate–polyacrylamide gel electrophoresis (SDS‐PAGE) was performed to separate the protein samples, which were transferred to polyvinylidene difluoride membranes (Millipore). The membranes were blocked in 5% (w/v) skim milk for 1 hr at room temperature and then incubated under gentle shaking at 4°C overnight with anti‐Parkin antibody (1:1,000, CST, #2132), anti‐α‐syn antibody (1:1,000, CST, Cat. #4179), anti‐P‐α‐syn antibody (1:5,000, Abcam, #ab51253), anti‐Ubiquitin antibody (1:2,000, Abcam, Cat. #ab7780), anti‐CD3δ antibody (1:1,000, CST, #99940), anti‐PLK2 antibody (1:1,000, CST, #14812), anti‐Cleaved Caspase‐3 antibody (1:1,000, CST, #9661), anti‐Cleaved PARP antibody (1:1,000, CST, #94885), and anti‐β‐actin (1:1,000, Bioss, Cat. #bs‐0061R). The membranes were then incubated with secondary antibodies for 1 hr at room temperature. Electrochemiluminescence reagents (Bio‐Rad) were used to display immunoblot signals, and ImageJ analysis software was used to measure band density. β‐Actin was used as a loading control. Each experiment was performed at least three times.

### 2.6 Coimmunoprecipitation

With the protease inhibitors, protein samples were extracted in RIPA buffer. The mixtures were treated with anti‐Parkin antibody (1:50, Abcam, Cat. #ab77924) or control IgG (Beyotime, Cat. # A7016) at 4°C overnight followed by incubation with Protein A/G (Beyotime, Cat. #P2012) at 4°C for 2 hr. After the samples were centrifuged at 1,500 *g* for 5 min, the supernatant was removed. Subsequently, the beads were washed three times with PBS to remove the proteins. Western blot analyses were then performed using anti‐α‐syn antibody (1:1,000, CST, Cat. #4179).

### 2.7 Virus transfection in cells

The Parkin gene lentivirus was purchased from the GK Genes Company. LV‐NC was used as the control virus. According to preliminary experiments, we determined the optimal infection conditions: virus concentration 1 × 10^7^ Tu/ml for 48 hr. SH‐SY5Y cells were infected with LV‐Parkin or LV‐NC at approximately 80% density for 48 hr. Then, the cells were treated with METH or PBS for the following experiment.

### 2.8 Virus transfection in mice

Mice were randomly divided into four groups (*n* = 3/group): an LV‐NC group, an LV‐Parkin group, an LV‐NC + METH group, and an LV‐Parkin + METH group. A stereotaxic injection protocol was performed as previously described by our laboratory (Xu et al., 2017; Zhu et al., 2018). Mice were anesthetized by 1% pentobarbital and fixed in a stereotaxic frame (Domitor). A total of 2 µl of LV‐NC or LV‐Parkin was injected into the right striatum at an injection rate of 1 µl/min. The specific coordinates were as follows: The bite bar was set at zero, 0.38 mm rostral to Bregma, 1.78 mm right lateral to the midline, and 3.25 mm ventral to the dura. The striatum was selected because it was the major target site for METH according to our previous study (Chang et al., 2005). Mice were exposed to METH or saline after 2 weeks.

### 2.9 Statistical analysis

All data are presented as the mean ± standard deviation (*SD*). SPSS version 20.0 (IBM Corporation) was used to perform all data analysis. Comparison between groups was conducted by one‐way ANOVA followed by the Bonferroni post hoc analysis or the Mann–Whitney *U* test for two independent samples. *p* < .05 was considered statistically significant. Details on the statistics are shown in Table 1. Each experiment was repeated three times.

**Table 1:** Details on the statistics

Figure	Group	Protein	*F*	Sig.
Figure 1	–	Alpha‐syn	15.898	0.000
	–	Monoubiquitin	5.010	0.010
	–	Polyubiquitin	84.278	0.000
Figure 2	Midbrain	Monoubiquitin	–	0.196
		Polyubiquitin	–	0.003
	Striatum	Monoubiquitin	–	0.220
		Polyubiquitin	–	0.004
Figure 3	Time	Parkin	6.071	0.005
		Alpha‐syn	8.528	0.001
	Concentration	Parkin	5.984	0.005
		Alpha‐syn	7.182	0.003
		Co‐ip	6.457	0.004
Figure 4	Midbrain	Parkin	–	0.012
		Alpha‐syn	–	0.003
	Striatum	Parkin	–	0.015
		Alpha‐syn	–	0.001
Figure 6	SH‐SY5Y	Alpha‐syn	21.576	0.000
		PARP	25.165	0.000
		P‐α‐syn	15.108	0.001
		Caspase‐3	22.273	0.000
		PLK2	41.008	0.000
		CD3δ	49.042	0.000
	Midbrain	Alpha‐syn	23.316	0.000
		PARP	24.835	0.000
		P‐α‐syn	14.769	0.001
		Caspase−3	23.235	0.000
		PLK2	40.849	0.000
		CD3δ	33.298	0.000
	Striatum	Alpha‐syn	25.792	0.000
		PARP	18.165	0.001
		P‐α‐syn	26.636	0.000
		Caspase‐3	40.082	0.000
		PLK2	35.773	0.000
		CD3δ	31.984	0.000

## 3 RESULTS

### 3.1 MG132 (a proteasome inhibitor) increases the expression of α‐syn and polyubiquitin in SH‐SY5Y cells

**Figure 1:**
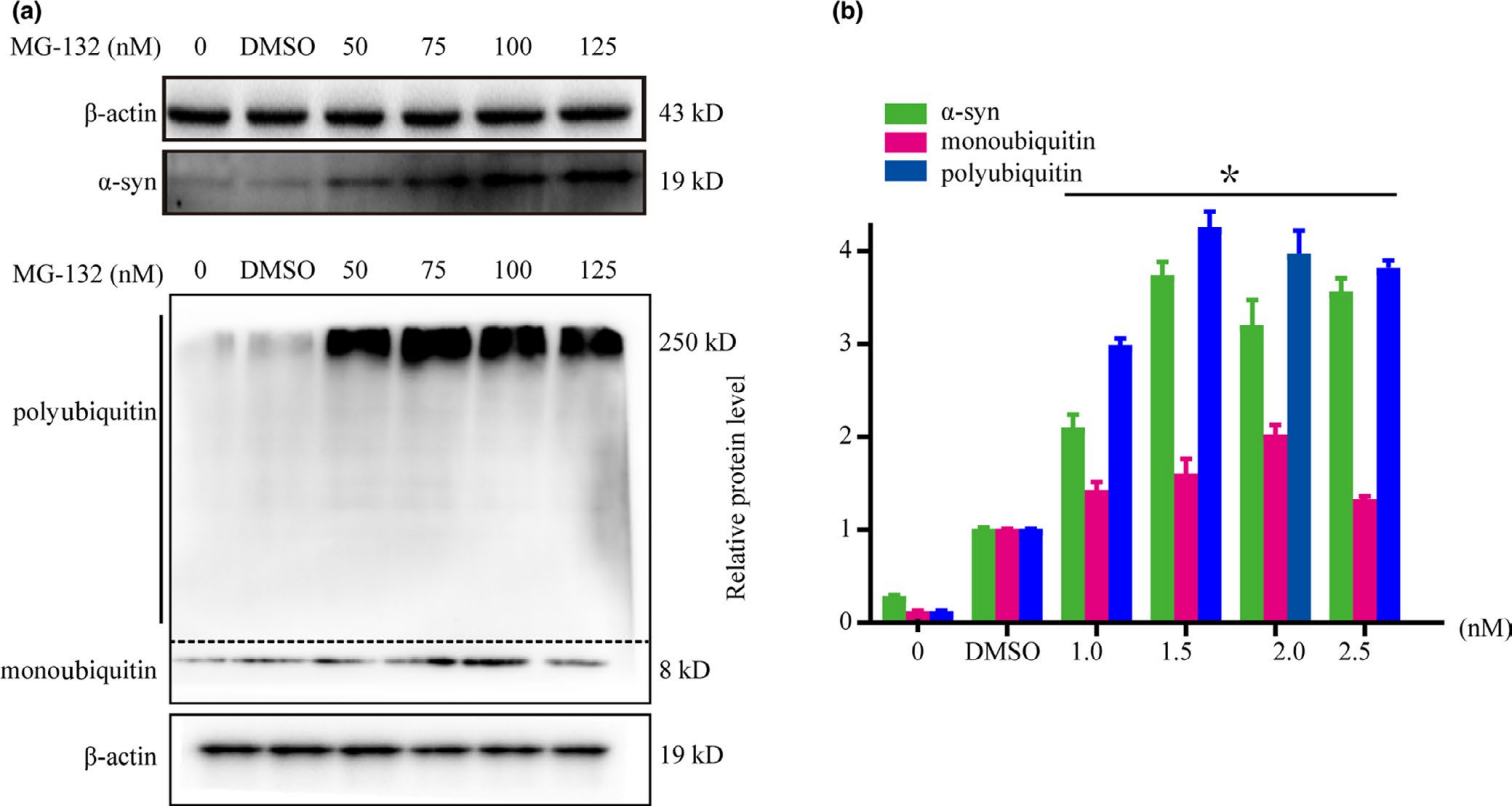
MG132 (proteasome inhibitor) increases the levels of alpha‐synuclein (α‐syn) and polyubiquitin in SH‐SY5Y cells. SH‐SY5Y cells were exposed to a range of MG132 doses for 24 hr. Western blot (a) and quantitative analyses (b) showed that MG132 increased α‐syn and polyubiquitin expression in SH‐SY5Y cells. Western blot (a) and quantitative analyses (b) were performed to determine the levels of α‐syn and ubiquitin. β‐actin was used as a loading control. *p < .05 compared with the control group. The data were analyzed using one‐way ANOVA followed by the Bonferroni post hoc analysis. DMSO was used as the drug carrier at a concentration of <0.2%. Each experiment was repeated three times

To test the role of the UPS pathway in α‐syn degradation, we treated SH SY5Y cells with different concentrations of ubiquitin proteasome inhibitor MG132 (0–125 nM) cells for 24 hr. Western blot results showed that the levels of α‐syn and polyubiquitin were significantly increased after exposure to MG132 (Figure 1a,b). The results suggest that impairment of the UPS pathway may be of great importance to α‐syn degradation dysfunction.

### 3.2 METH increases polyubiquitin protein expression in vitro and in vivo

**Figure 2:**
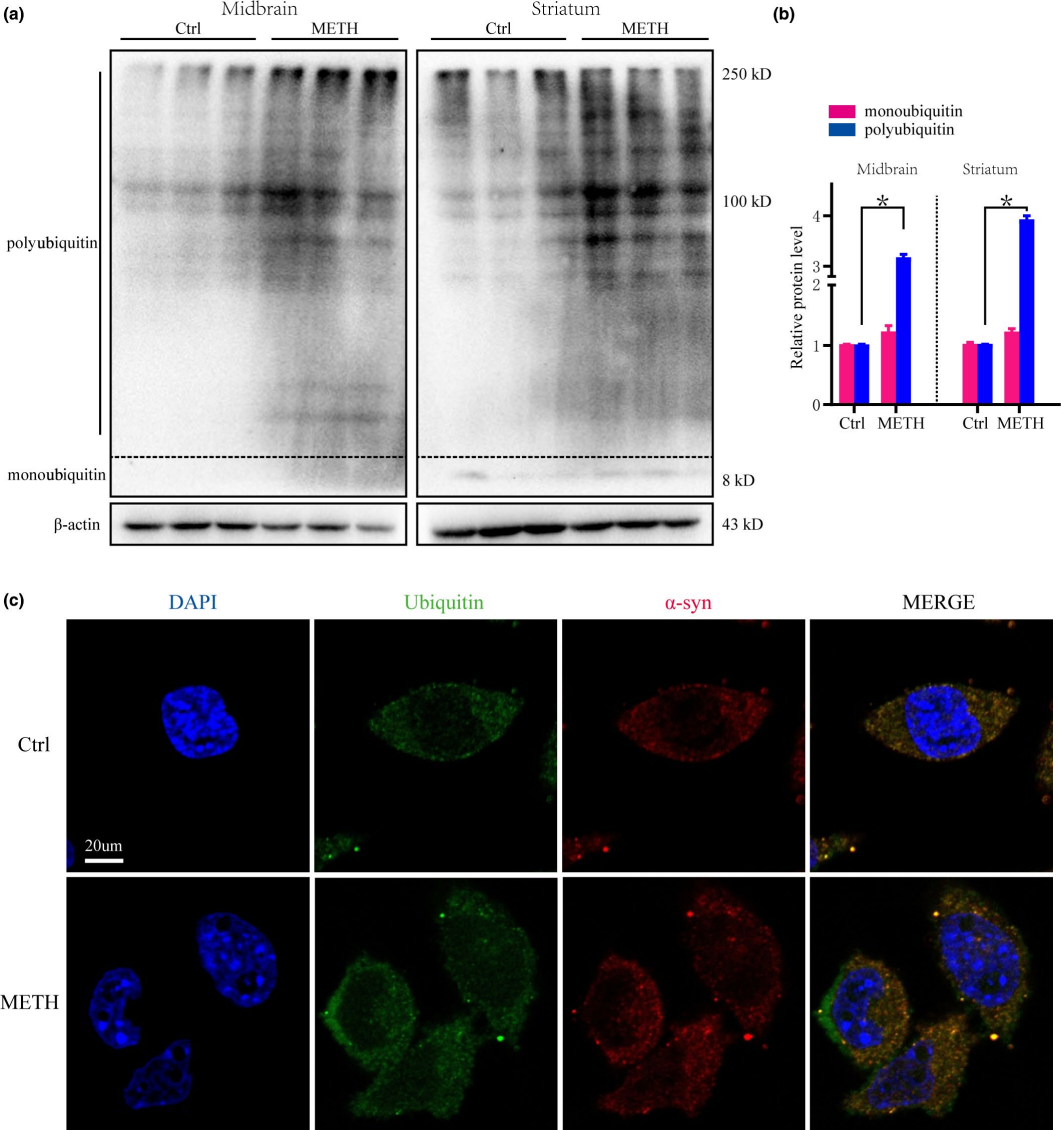
Methamphetamine (METH) increases polyubiquitin protein expression in vitro and in vivo. Mice were randomly divided into two groups: a control group and a subacute METH group (*n* = 3/group). Western blot (a) and quantitative analyses (b) showed that METH increased polyubiquitin expression in the midbrain and striatum of male C57 mice. SH‐SY5Y cells were exposed to 2 mM METH for 24 hr. Fluorescence microscopy (c) results showed ubiquitin and α‐syn were colocalized and expressed at higher levels in METH‐treated SH‐SY5Y cells than in control cells. Ubiquitin was stained with an anti‐Ubiquitin antibody (green), α‐syn was stained with an anti‐α‐syn antibody (red), and nuclei were counterstained with DAPI (blue). β‐actin was used as a loading control. *p < .05 compared with the control group. The data were analyzed using the Mann–Whitney *U* test. Each experiment was repeated three times

To test the effect of ubiquitin on METH‐induced toxicity, we determined the levels of ubiquitin in vitro and in vivo. Western blot results showed no difference in monoubiquitin between the two groups. However, polyubiquitin protein expression in the midbrain and striatum was significantly higher in the METH exposure group than in the control group (Figure 2a,b). In addition, the immunofluorescence results showed that the levels of ubiquitin and α‐syn proteins were increased and exhibited colocalization after METH exposure (Figure 2c). Taken together, the results suggest that METH exposure can increase the level of polyubiquitin in vitro and in vivo.

### 3.3 METH Influences Parkin and α‐syn Protein Expression in vitro and in vivo

**Figure 3:**
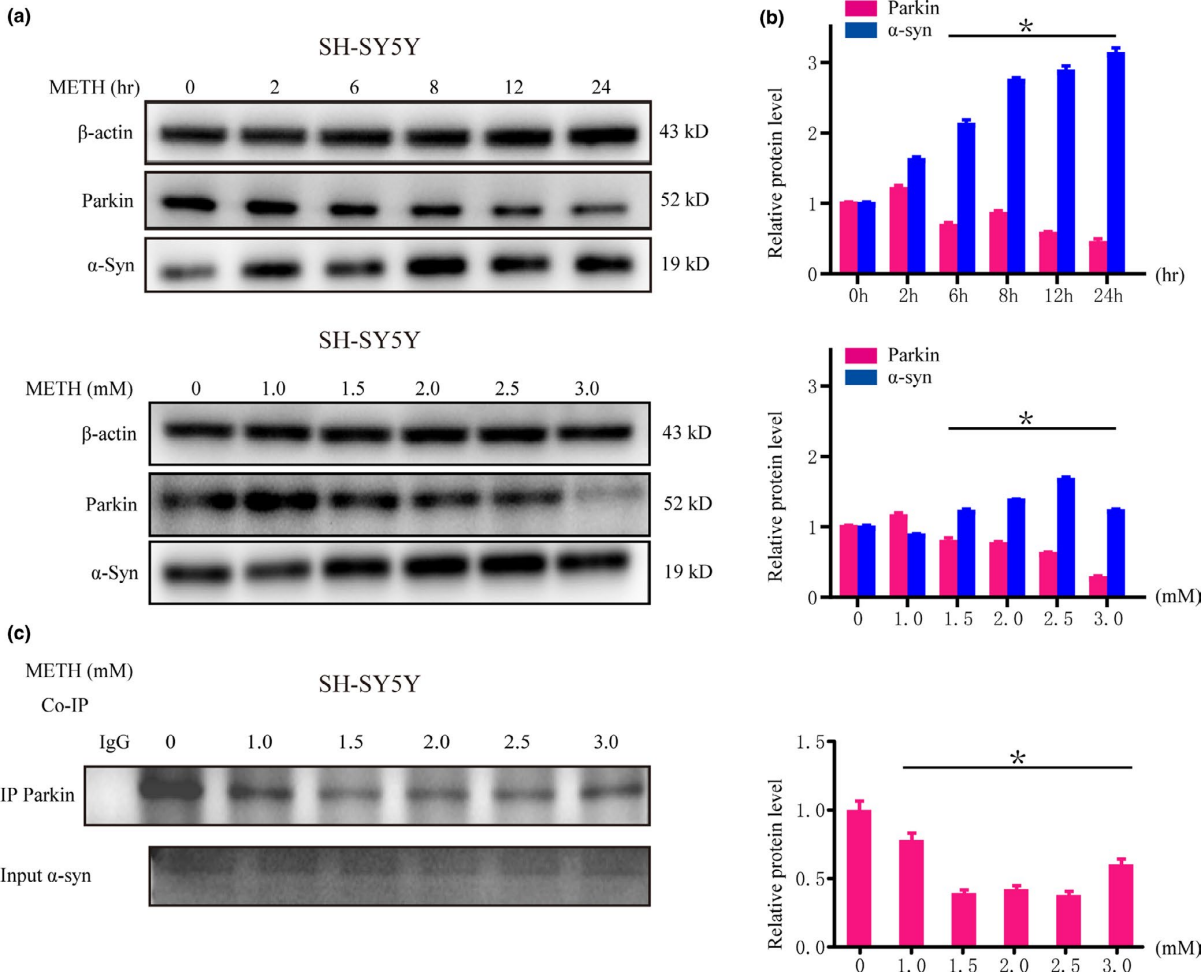
Methamphetamine (METH) increases α‐syn expression, decreases Parkin protein expression, and reduces the interaction between Parkin and α‐syn in SH‐SY5Y cells. SH‐SY5Y cells were treated with 0.5–3.0 mM METH for 24 hr or 2.0 mM METH for 2–24 hr. Western blot (a) and quantitative analyses (b) showed that METH increased the expression of α‐syn and decreased the expression of Parkin in dose‐ and time‐dependent manners. Coimmunoprecipitation results (c) showed that the interaction between Parkin and α‐syn was reduced after METH exposure. Cells were immunoprecipitated with an anti‐Parkin antibody and then with an anti‐α‐syn antibody and analyzed with Western blot. IgG was used as a negative control, and β‐actin was used as a loading control. *p < .05 compared with the control group. The data were analyzed using one‐way ANOVA followed by the Bonferroni post hoc analysis. Each experiment was repeated three times

To determine the effects of METH on Parkin and α‐syn proteins, we treated SH‐SY5Y cells with different doses of METH for 24 hr or 2.0 mM METH for 2–24 hr. Western blot results revealed that Parkin showed a trend of first increasing and then decreasing in a dose‐dependent and time‐dependent manner after METH exposure (Figure 3a,b). For example, Parkin level was increased at 2 hr and 1.0 mmol and then decreased with time and concentration. Furthermore, the level of α‐syn protein increased in a dose‐dependent and time‐dependent manner after METH exposure (Figure 3a,b).

**Figure 4:**
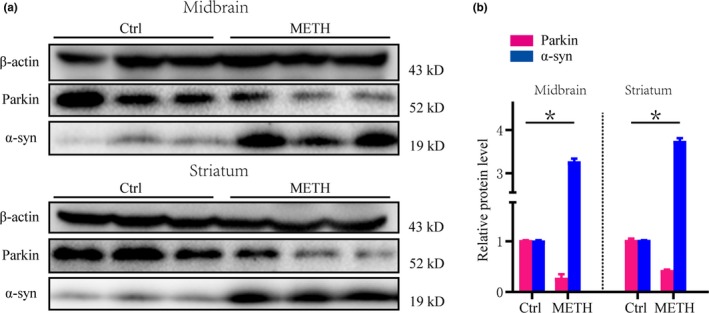
Methamphetamine (METH) increases α‐syn expression and decreases Parkin protein expression in the midbrain and striatum of mice. Mice were randomly divided into two groups: a control group and a subacute METH group (*n* = 3/group). Western blot (a) and quantitative analyses (b) showed that METH increased α‐syn expression and decreased Parkin protein expression in vivo. β‐Actin was used as a loading control. *p < .05 compared with the control group. The data were analyzed using the Mann–Whitney *U* test. Each experiment was repeated three times

To verify the results obtained in vitro, we analyzed Parkin and α‐syn protein expression in the midbrain and striatum of mice. The Western blot results indicated that in the subacute exposure group, Parkin and α‐syn protein expression in the midbrain and striatum was increased (Figure 4a,b). These results are consistent with the results in vitro.

The results presented above suggest that the levels of α‐syn and Parkin protein were affected by METH exposure. Moreover, the coimmunoprecipitation results showed that the interaction between Parkin and α‐syn was decreased in SH‐SY5Y cells after METH exposure (Figure 3c).

### 3.4 The increase in α‐syn induced by METH is relieved when Parkin is overexpressed

**Figure 5:**
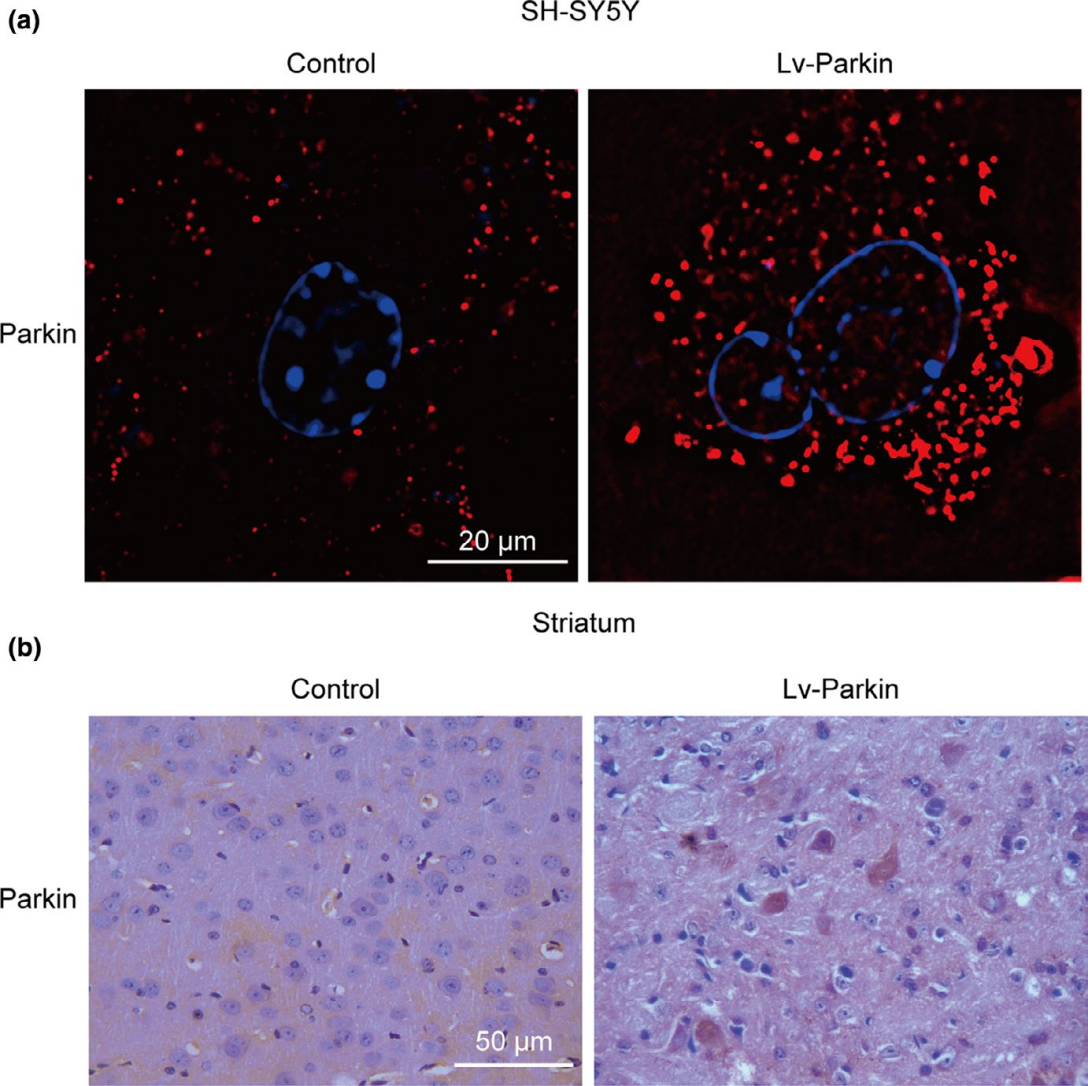
Parkin was overexpressed after the transfection of LV‐Parkin in SH‐SY5Y cells and C57 mice. SH‐SY5Y cells and male C57 mice were transfected with LV‐NC or LV‐Parkin (*n* = 3/group). Immunofluorescence (a) and immunohistochemistry analyses (b) showed that the distribution and extent of Parkin were significantly increased in cells and striatum transfected with LV‐Parkin. Parkin was stained with an anti‐Parkin antibody (red), and nuclei were counterstained with DAPI (blue). Each experiment was repeated three times

**Figure 6:**
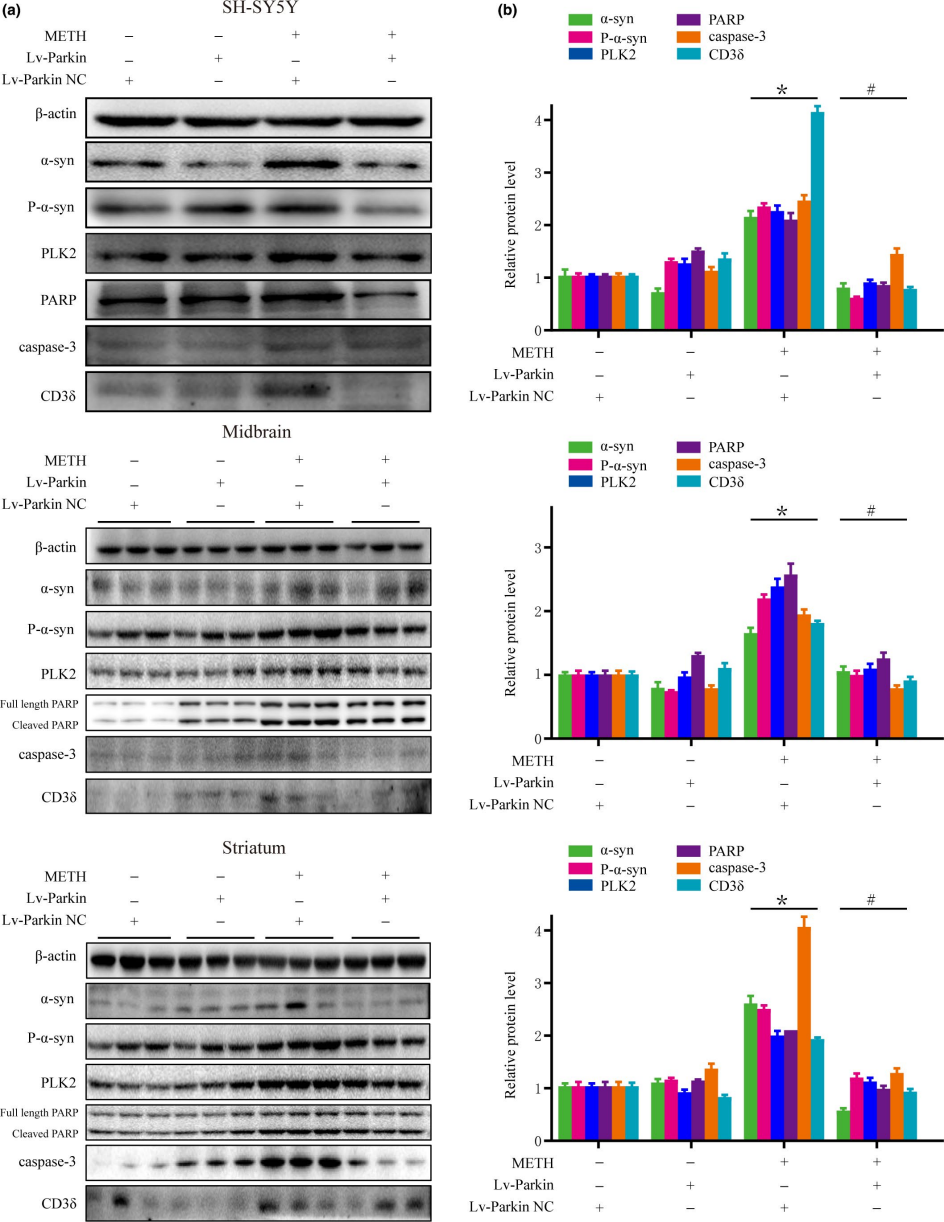
The increase in α‐syn induced by METH is relieved when Parkin is overexpressed. SH‐SY5Y cells and male C57 mice were transfected with LV‐NC or LV‐Parkin (*n* = 3/group). Then, cells and mice were treated or not with METH. Western blot (a) and quantitative analyses (b) showed that the levels of α‐syn, P‐α‐syn, PLK2, PARP, Caspase‐3, and CD3δ in the groups treated with LV‐NC + METH were significantly increased relative to the corresponding levels in the LV‐NC + Saline groups. These increases were mitigated to large extent after transfection with LV‐Parkin. β‐Actin was used as a loading control. *p < .05 compared with the LV‐NC + Saline group; #*p* < .05 compared with the LV‐NC + METH group. The data were analyzed using one‐way ANOVA followed by the Bonferroni post hoc analysis. Each experiment was repeated three times

To investigate the effect of Parkin on METH‐induced α‐syn degradation, we used LV‐Parkin to overexpress Parkin in SH‐SY5Y cells and mice. The level of Parkin protein was significantly increased after virus injection (Figure 5a,b). In the saline‐exposed group, there was no difference in the levels of α‐syn, P‐α‐syn, PLK2, PARP, Caspase‐3, and CD3δ between the LV‐NC and LV‐Parkin groups. However, after exposure to METH, the levels of α‐syn, P‐α‐syn, PLK2, PARP, Caspase‐3, and CD3δ were increased. More importantly, transfection with LV‐Parkin relieved these increases (Figure 6a,b).

The results were verified in mice. We injected LV‐Parkin or LV‐NC into the right striatum of mice to verify the effect of Parkin on the degradation of α‐syn induced by METH. The results showed that the levels of α‐syn, P‐α‐syn, PLK2, PARP, Caspase‐3, and CD3δ in the midbrain and striatum of mice treated with LV‐NC + METH were increased significantly relative to the corresponding levels in the LV‐NC + Saline group (Figure 6a,b). In addition, the increases were mitigated to large extent after transfection with LV‐Parkin (Figure 6a,b). The results of in vivo and in vitro experiments revealed that the increases in α‐syn and α‐syn degradation dysfunction induced by METH can be relieved when Parkin is overexpressed.

## 4 DISCUSSION

In this study, we found that Parkin level decreased and α‐syn level increased after exposure to METH in vitro and in vivo. Furthermore, we found that polyubiquitin was increased by METH and MG132, which suggests that impairment of the UPS pathway may contribute to the dysfunction of α‐syn degradation after METH exposure. The reason for the increase in polyubiquitin may have been that Parkin, as an E3 ubiquitin ligase that regulates the level of α‐syn ubiquitination, decreased after exposure to METH. When Parkin is overexpressed, proteasome activity increases, and α‐syn levels and METH‐induced apoptosis are significantly reduced. Our results and previous studies (Liu et al., 2019) show that Parkin has an important role in the METH‐induced dysfunction of α‐syn degradation.

α‐syn is a neuroprotein widely expressed in the brain and is involved in various neurodegenerative diseases (Al‐Mansoori, Hasan, Al‐Hayani, & El‐Agnaf, 2013; Melki, 2015). Studies have reported that various structural forms of pathological state α‐syn, such as fibrils, fibers, oligomers, and amorphous oligomers, and their posttranslational modifications, such as phosphorylation, nitration, and ubiquitination, are neurotoxic (Chen & Feany, 2005; Cremades et al., 2012). There is a dynamic balance between normal and misfolded α‐syn under physiological conditions (Schaser et al., 2019). The balance is broken when cells are exposed to oxidative stress, poison, and other stress conditions. Fibrils rapidly aggregate into insoluble macromolecules to form LBs (Auluck, Chan, Trojanowski, Lee, & Bonini, 2002). In the study, we verified that the level of α‐syn increases with increasing METH concentration and exposure time, which is consistent with previous studies (Qiao et al., 2019; Zhu et al., 2018). The accumulation of α‐syn can lead to apoptosis and even death of neurons. Most misfolded, damaged, and incompletely assembled proteins in the body are cleared by the UPS (Cai et al., 2019; Yuan et al., 2019). However, our results indicate that polyubiquitin is increased after METH exposure, as it is after MG132 exposure, which suggests impairment of the UPS in degrading α‐syn. In the subacute METH exposure model, proteasome activity is decreased; thus, the degradation of ubiquitinated proteins decreases, which leads to an increase in polyubiquitination. We propose the hypothesis that Parkin is of great significance to the METH‐induced dysfunction of α‐syn degradation.

Parkin functions as an E3 ubiquitin ligase in degrading proteins of the UPS. The function of Parkin is impaired when stimulated by external stimuli such as drugs (Moszczynska & Yamamoto, 2011). As a result of this impaired functioning, substrate proteins are not fully degraded, and they consequently accumulate in cells. Studies have shown that Parkin specifically recognizes α‐syn and promotes its degradation (Liu et al., 2019). Our study found that the level of Parkin showed a trend of first increasing and then decreasing in SH‐SY5Y cells in a dose‐dependent and time‐dependent manner after METH exposure. A possible reason for this pattern is that Parkin undergoes a compensatory increase and then decreases with increasing exposure time and METH concentration. METH increases α‐syn levels in a dose‐ and time‐dependent manner. In addition, the coimmunoprecipitation results showed that the interaction between Parkin and α‐syn was reduced significantly by METH exposure. The results suggest that Parkin is of great significance to the dysfunction of α‐syn degradation induced by METH.

The study also revealed that the serine 129‐site phosphorylation modification of α‐syn (P‐α‐syn) increased significantly in METH‐treated cells. Studies have shown that under physiological conditions, <4% of α‐syn in brain neurons is in phosphorylated α‐syn forms, whereas more than 90% α‐syn is phosphorylated in LBs (Anderson et al., 2006; Fujiwara et al., 2002; Oueslati, 2016). Our results confirm that METH induces an increase in the α‐syn phosphorylation in SH‐SY5Y cells, which is of great significance to the development of METH neurotoxicity. According to the literature, CD3δ is the most characteristic substrate for ER‐related degradation. When E3 ligase cannot degrade CD3δ, the protein level of CD3δ is increased, and the stability is greatly improved. CD3δ is closely related to proteasome activity (Garcillan et al., 2014). The results showed the CD3δ was increased after METH exposure, indicating that proteasome activity was reduced after METH treatment. Such reduction could result in the lack of degradation of α‐syn, which then accumulates in cells. Furthermore, compared with the environment of cells in brain tissue, the environment of cells cultured in vitro is simple, and the response to stimulation is large. When cells are stimulated, the microenvironment around the cells has a buffer effect, which may lead to the degradation of CD3d. There is an inhibitory effect on its increase, so the increase in CD3d in the brain is not as large as it is in cells (Seo et al., 2019). Apoptosis is an autonomous procedure of cell death that is of great significance to the development of the nervous system and to maintaining cellular homeostasis. Many studies have reported that neuronal apoptosis occurs during the development of neurodegenerative diseases such as AD, PD, and ALS (Ghavami et al., 2014). In this study, we found that the expression of the apoptosis‐related proteins Cleaved Caspase‐3 and Cleaved PARP was increased significantly after METH exposure, which indicated that apoptosis was increased by METH. These results are consistent with previous studies (Du et al., 2018; Xu et al., 2018).

In the present study, we overexpressed Parkin protein by transfection of human Parkin gene recombinant lentivirus in vitro and in vivo. Compared with LV‐NC + METH, the LV‐Parkin + METH group exhibited a significant decrease in α‐syn, demonstrating that Parkin can promote the degradation of α‐syn. Increased P‐α‐syn is an important pathological feature of PD, and α‐syn phosphorylation produces many insoluble oligomers. The phosphorylation modification at this site is mainly catalyzed by PLK2. Compared with the corresponding levels in the LV‐NC + METH group, PLK2 was significantly decreased in METH‐treated cells and mice, and P‐α‐syn was significantly reduced. In addition, the LV‐Parkin + METH group had higher proteasome activity and significantly reduced apoptosis relative to the LV‐NC + METH group. These results indicate that the overexpression of Parkin protein can promote the degradation of α‐syn and alleviate the neurotoxicity induced by METH, which is consistent with previous studies (Liu, Traini, Killinger, Schneider, & Moszczynska, 2013).

In summary, we verified that Parkin, as a E3 ubiquitin ligase, plays a critical role in METH‐induced dysfunction of α‐syn degradation in vitro and in vivo. The overexpression of Parkin significantly promoted the degradation of α‐syn and relieved METH‐induced neurotoxicity. It is necessary to study the mechanisms underlying the neuroprotective effect of Parkin under METH exposure and the role of the UPS pathway in α‐syn degradation after METH exposure.

## CONFLICT OF INTEREST

None declared.

## AUTHOR CONTRIBUTIONS

Yunle Meng and Honghua Qiao carried out the in vitro and in vivo experiments and performed the data analysis. Jiuyang Ding assisted with the in vitro experiments. Yitong He, Haoling Fan, and Chen Li assisted with the in vivo experiments. Pingming Qiu designed the study and reviewed the manuscript.

## Data Availability

The data supporting the results of this study are publicly available.
